# Photosynthetic activity in both algae and cyanobacteria changes in response to cues of predation

**DOI:** 10.3389/fpls.2022.907174

**Published:** 2022-07-25

**Authors:** Małgorzata Grzesiuk, Barbara Pietrzak, Alexander Wacker, Joanna Pijanowska

**Affiliations:** ^1^Department of Hydrobiology, Faculty of Biology, Institute of Functional Biology and Ecology, University of Warsaw Biological and Chemical Research Centre, Warszawa, Poland; ^2^Department of Biochemistry and Microbiology, Institute of Biology, Warsaw University of Life Sciences (SGGW), Warszawa, Poland; ^3^Department of Ecology and Ecosystem Modelling, Institute of Biochemistry and Biology, University of Potsdam, Potsdam, Germany; ^4^Department of Animal Ecology, Zoological Institute and Museum, University of Greifswald, Greifswald, Germany

**Keywords:** phytoplankton, grazing, predation, *Daphnia*, phenotypic plasticity, biotic stress, photosystem, PAM

## Abstract

A plethora of adaptive responses to predation has been described in microscopic aquatic producers. Although the energetic costs of these responses are expected, with their consequences going far beyond an individual, their underlying molecular and metabolic mechanisms are not fully known. One, so far hardly considered, is if and how the photosynthetic efficiency of phytoplankton might change in response to the predation cues. Our main aim was to identify such responses in phytoplankton and to detect if they are taxon-specific. We exposed seven algae and seven cyanobacteria species to the chemical cues of an efficient consumer, *Daphnia magna*, which was fed either a green alga, *Acutodesmus obliquus*, or a cyanobacterium, *Synechococcus elongatus* (kairomone and alarm cues), or was not fed (kairomone alone). In most algal and cyanobacterial species studied, the quantum yield of photosystem II increased in response to predator fed cyanobacterium, whereas in most of these species the yield did not change in response to predator fed alga. Also, cyanobacteria tended not to respond to a non-feeding predator. The modal qualitative responses of the electron transport rate were similar to those of the quantum yield. To our best knowledge, the results presented here are the broadest scan of photosystem II responses in the predation context so far.

## Introduction

The ability to sense and adequately respond to a predator’s presence is vital to survival. In aquatic environments, waterborne chemical cues released by predators elicit responses in prey species across functional groups (phytoplankton, zooplankton, nekton, benthos, etc.) and across taxa: in animals—from bdelloid rotifers to amphibians (e.g., Chivers et al., 2015; Parysek and Pietrzak, 2021), in protists—from hyptophytes to myzozoans and brown algae (e.g., Long et al., 2007; Selander et al., 2011; Harvey and Menden-Deuer, 2012), in green algae (e.g., Fisher et al., 2016), and in prokaryotes (e.g., Yang et al., 2009).

Phytoplankton species of different taxa have developed various defenses against the herbivores that prey on them (e.g., review Van Donk et al., 2011; Pančić and Kiørboe, 2018; Lürling, 2021). These defenses may be active at various stages of the encounter with the herbivorous predators (both as herbivores feeding on plants and as predators killing the consumed organism). *Ceratium* sp. and *Staurastrum* sp. are not swallowed by these planktonic predators thanks to the outgrowths that increase their size and complicate their shape. Green algae *Acutodesmus* (formerly *Scenedesmus*), *Pediastrum*, and *Volvox* form colonies and coenobia. This makes them too large for planktonic herbivores to feed on them (e.g., Lürling, 2003; Rojo et al., 2009). A similar phenomenon can be observed in the cyanobacteria *Aphanizomenon flos-aquae*, which form large trichomes, thus forming inedible clusters (e.g., Cerbin et al., 2013). In addition to morphological changes, chemical mechanisms by which planktonic producers defend themselves against herbivores are also known. For example, cyanobacteria *Microcystis aeruginosa* produce toxins in response to herbivory of planktonic animals (Jang et al., 2003). Green algae *Sphaerocystis schroeteri* produce gelatinous sheaths resistant to *Daphnia* digestive enzymes, so they can pass through the gastrointestinal tract of the herbivores without any damage, or even acquire additional nutrients and use them for enhanced growth (Porter, 1976).

Activating a defense mechanism requires increased production or, otherwise, limits energy available for other life processes (Van Donk et al., 2011; Pančić and Kiørboe, 2018; Lürling, 2021). Generating additional metabolic energy may involve modifying photosynthesis, a biochemical process that converts light energy into chemically bonded energy. Phytoplankton species capable of generating and using additional energy to defend themselves against herbivores may gain an advantage over species that have not developed such a mechanism. However, the effects of predator presence on photosynthetic activity have hardly been reported so far. Recently, Albini et al. (2019) showed that both photosystem II (PSII) efficiency and cellular chlorophyll *a* content decreased significantly during *A. obliquus* colony formation, interpreted as the cost thereof. However, an increased energetic demand in the presence of herbivores may require a periodic increase in photosynthetic efficiency. Therefore, the main aim of this study was to acquire information on how the photosynthetic efficiency in phytoplankton cells of different taxa changes in response to predation cues.

Defense against herbivores, e.g., colony formation, can be induced by chemical information about their presence (e.g., Hessen and Van Donk, 1993), yet, in most studies phytoplankton was simply grown with the herbivore and the nature of the chemical cues has not been further elucidated. From some studies, we know that either “alarm” cues, released from injured phytoplankton cells (Senft-Batoh et al., 2015), or the presence of kairomones—substances released by the herbivorous predators themselves, irrespective of their current diet (Yokota and Sterner, 2011; Wejnerowski et al., 2018)—were enough to elicit a response in the respective producer. Each of these cues provides a somewhat different information about the environment and might demand both different perception pathways and different responses. We thus hypothesized that the presence of “feeding-related” chemical cues, i.e., of a predator feeding on either prokaryotic or eukaryotic producers (kairomone and alarm cues), or of the predator not feeding at all (kairomone alone), each elicits a different response in different phytoplankton species, with the weakest response to the non-feeding predator.

## Materials and methods

### Experimental organisms

In our experiments, we tested seven algae and seven cyanobacteria species (Table 1) of either unicellular or filamentous morphology. We chose species commonly found in freshwater ecosystems co-occurring with herbivores such as *Daphnia*.

**TABLE 1:** List of tested algae and cyanobacteria.

Species	Strain number	Class	Morphology
*Acutodesmus obliquus*	SAG 276-3a	Chlorophyceae	unicellular
*Chlamydomonas reinhardtii*	SAG 11-32b	Chlorophyceae	unicellular
*Chlorella vulgaris*	SAG 211-11b	Chlorophyceae	unicellular
*Cryptomonas* sp.	SAG 26-80	Cryptophyceae	unicellular
*Nannochloropsis limnetica*	SAG 18.99	Eustigmatophyceae	unicellular
*Oedogonium cardiacum*	SAG 575-1b	Chlorophyceae	filamentous
*Oedogonium* sp.	SAG 54.94	Chlorophyceae	filamentous
*Anabaena flos-aquae*	SAG 30.87	Cyanophyceae	filamentous
*Cylindrospermopsis raciborskii*	ZIE11*	Cyanophyceae	filamentous
*Nostoc ellipsosporum*	SAG 1453-2	Cyanophyceae	unicellular
*Planktothrix agardhii*	NIVA-CYA 34	Cyanophyceae	filamentous
*Uronema minuta*	SAG 386-1	Cyanophyceae	filamentous
*Synechococcus elongatus*	SAG 89.79	Cyanophyceae	unicellular
*Synechocystis aquatilis*	SAG 90.79	Cyanophyceae	unicellular

*Wiedner et al. (2007).

All cyanobacteria and algae were obtained from cultures maintained in the Institute for Biochemistry and Biology, University of Potsdam, Germany. They were cultured in Erlenmeyer flasks in WC medium (pH buffered with Hepes and adjusted to 7.0) at 20°C, and gently mixed daily. Light intensity at the surface of the flasks was 120 μmol m^–2^ s^–1^ and was supplied in a 16:8 light:dark cycle. The medium was renewed regularly to maintain a high growth rate in the cultures by excluding nutrient limitation while maintaining low algal density (and thus also excluding light limitation) and stable pH conditions. To control for potential nutrient limitation, before the experiments, the vital condition of all cultures was verified by measuring nutrient-induced fluorescence transient (NIFT) responses (Shelly et al., 2010; Spijkerman et al., 2016; see Supplementary Figure 1 for details). For inorganic phosphate (P_i_) addition, we used a final concentration of 10 μM KH_2_PO_4_. Spikes of nitrate were used at a final concentration of 0.1 mM NaNO_3_. None of the cultures were limited by either P_i_ or N (Supplementary Figures 1B,C).

The filter feeder *Daphnia magna* was the chosen predator, for being an extremely effective consumer of phytoplankton within a broad spectrum of sizes (Gliwicz, 2003), even among other species in the genus, on the one hand, and a model organism widely used in evolutionary, ecological, and ecotoxicological studies (Colbourne et al., 2011; Lampert, 2011) on the other. We used *D. magna* from a clonal library maintained in the Institute for Biochemistry and Biology, University of Potsdam, Germany.

### Media preparation

We cultured *Daphnia magna* in ADaM medium (10–20 ind. L^–1^) under standard laboratory conditions (20°C and at a 16 h:8 h light dark cycle). As a food source, we used either the green alga *Acutodesmus obliquus* or the cyanobacterium *Synechococcus elongatus* in concentrations equivalent to 1 mg C_*org*_ L^–1^. To acquire the medium and its chemical cues, adult *Daphnia* females were exposed for at least 2 days to the above-mentioned food types. Then *Daphnia* was taken out and the remaining medium was centrifuged (5,000 × *g*, 5 min) to remove algae or cyanobacteria cells. An additional treatment was added for randomly selected eight of the studied species, in which they were exposed to *Daphnia* kept for 24 h without food supplementation to eliminate prey alarm cues. This way, three so-called “*Daphnia* media” were obtained, namely, *Daphnia* fed alga, fed cyanobacterium, or starved.

### Experiments

For testing the phytoplankton cells responses, five biological replicates of each algal or cyanobacterial culture were centrifuged (10 000 × *g*, 5 min) to isolate them from their culture medium. The cells were resuspended either in “*Daphnia* culture medium” (containing chemical cues), i.e., ADaM medium in which *Daphnia* was exposed, or in a control ADaM medium without contact with animals. In addition, to avoid the effect of nutrient limitation on the phytoplankton cells, we added 10 μM KH_2_PO_4_ and 0.1 mM NaNO_3_. Then, 2 ml of algal or cyanobacterial suspension was pipetted into a test tube and placed in the light (300 μmol photons m^–2^ s^–1^; light bulb Redium, HRI 250 W/D). To choose proper exposure time, we exposed selected species for 10, 60, 120, and 240 min in a pilot experiment. As already after 10 and 60 min a reaction of photosynthetic parameters was observed, we used these times in further experiments.

After the 10 and 60-min exposures (10’ and 60’), photosynthesis was instantly measured *via* rapid light curves using a Phyto-PAM fluorometer (Heinz Walz GmbH, Effeltrich, Germany). Photosynthetic activity was estimated by using saturation pulse quenching analysis in samples measured directly from the light. Rapid light curves consisted of ten actinic light intensities, increasing progressively: 4, 32, 64, 128, 192, 256, 320, 384, 448, 512 μmol m^–2^ s^–1^. From the first saturating flash, maximum fluorescence (*Fm’*) and fluorescence (*F’*) in light-adapted samples were recorded and the quantum yield of photosystem II was estimated as its (PSII) operating efficiency: (*Fm’-F’*)/*Fm’* (Baker, 2008). It represents the ratio of emitted and absorbed photons, which indicates photosynthetic efficiency (Marzetz et al., 2020). The fluorescence response to the different light intensities was fitted to the model of Eilers and Peeters (1988), and automatically (Phyto-Win Software V 1.45) was used to derive an approximate estimate of the maximum electron transport rate at light saturation (ETR_max_) (and the other photosynthetic parameters, Supplementary Tables 1A–E).

### Data analysis

As phytoplankton species differ in their absolute photosynthetic activity under control conditions, and as in this study we focus on the direction and strength of the impact of perceived predation treatment, we used effect sizes. Thus, to compare the effects of exposures across taxa and experiments, the effect sizes were calculated as Cohen’s d using the R package “effsize” (Torchiano, 2020) for each comparison against the control treatment. As a preliminary analysis showed no overall significant difference between the effects of 10’ and 60’ exposures, the data for the two-time exposures were pooled. Also, as the relative values over the four read channels changed with the treatment, to capture these responses, we used all four channels data and these were pooled, too (see also the model below). Group means and 95% CIs were calculated using the R package “rcompanion” (Mangiafico, 2020). The numbers of species that showed a significant decrease, no significant effect, or a significant increase in parameter values were counted for a summary presentation.

We next analyzed the magnitude of the absolute effects. To allow for the correlation of errors (four channels for each read) and unequal variances, we fitted a linear model to the absolute Cohen’s d data by maximizing the restricted log-likelihood using generalized least squares (GLS) with the R package “nlme” (Pinheiro et al., 2019). A type-II analysis-of-variance table was calculated for the model using the Anova function of the R package “car” (Fox and Weisberg, 2019). *Post hoc* Tukey’s comparisons were performed with the R package “multcomp” (Hothorn et al., 2008). All analyses were performed in R 4.1.2 language and environment (R Core Team, 2021).

## Results

**FIGURE 1:**
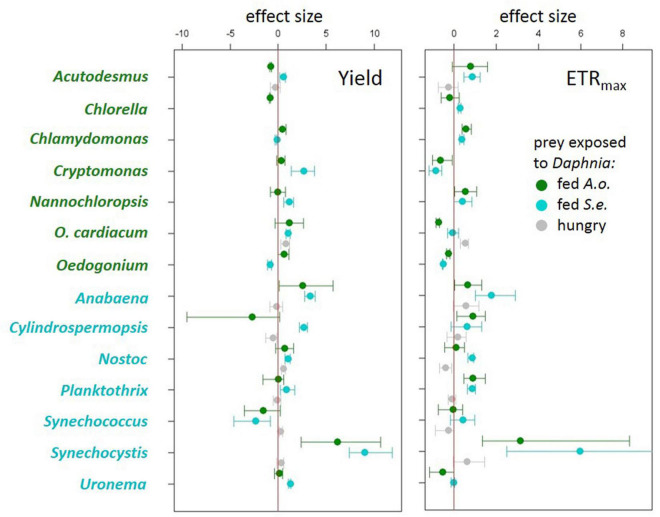
Effect sizes (Cohen’s d ± 95% confidence intervals) for each treatment to control comparison for quantum yield of photosystem II (left) and electron transport rate (ETRmax) (right) from experiments using algae (green species labels) or cyanobacteria (cyan species labels) as prey exposed either to *Daphnia* fed *Acutodesmus obliquus* (green circles), *Daphnia* fed *Synechococcus elongatus* (cyan circles) or hungry *Daphnia* (gray circles).

Algae and cyanobacteria from the tested species showed distinct photosynthetic responses depending on the chemical cues provided by the herbivore fed different diets. In most algae and cyanobacteria species the yield of PSII (estimated as its operating efficiency) and the estimated ETR_*max*_ increased when they were exposed to chemical cues of *Daphnia* previously feeding on cyanobacterium (Figure 1, cyan circles, summary results in Table 2), whereas in most of these species yield did not change in response to chemical cues of *Daphnia* fed alga (Figure 1, green circles, summary results in Table 2). Altered yields and ETR_*max*_ estimates were hardly observed if algae or cyanobacteria were exposed to chemical cues from starved daphnids (Figure 1, gray symbols, summary results in Table 2). The raw yield and ETR_*max*_ estimation data are provided in Supplementary Tables 1A–E.

**TABLE 2:** Numbers of tested species in which quantum yield of photosystem II or electron transport rate, ETRmax, significantly decreased (–), did not change (0), or significantly increased (+) in the presence of the chemical cues of *Daphnia* fed different types of diet (green alga, cyanobacterium, or starved).

Parameter		Yield						ETRmax					
	Prey	Algae			Cyanobacteria			Algae			Cyanobacteria		
		
*Daphnia* diet	Effect	–	0	+	–	0	+	–	0	+	–	0	+
green alga		2	5	–	–	5	2	3	3	1	1	2	4
cyanobacterium		1	2	4	1	–	6	1	3	3	–	3	4
starved		–	1	1	1	4	1	–	1	1	1	5	–

**FIGURE 2:**
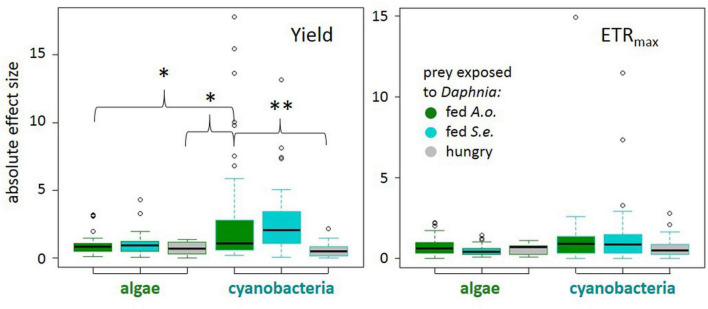
Absolute effect sizes (|Cohen’s d| : median, quartiles, and 1.5 interquartile distance) for each treatment to control comparison for quantum yield of photosystem II (left) and electron transport rate (right) by prey taxon (algae or cyanobacteria) and predator treatment: *Daphnia* fed *Acutodesmus obliquus* (green boxes), *Daphnia* fed *Synechococcus elongatus* (cyan boxes), or hungry *Daphnia* (gray boxes). Significant differences at *p* < 0.01 (**) or *p* < 0.05 (*) (see model details in text).

The magnitude of phytoplankton responses, i.e., effect size calculated as Cohen’s d, was dependent on several factors: (1) the phytoplankton cells exposure to chemical cues from daphnids in the “experimental medium” and (2) the taxon of phytoplankton tested (ANOVA on GLS results, experimental medium: χ^2^_1_ = 6.94, *p* = 0.0312; experimental medium × taxon: χ^2^_1_ = 11.1, *p* = 0.0039, Supplementary Table 2). In particular, cyanobacteria responses in quantum yield to chemical cues from *Daphnia* fed with green alga were larger than those to chemical cues from hungry *Daphnia* (Figure 2). If exposed to a medium from *Daphnia* fed with a green alga, the response of cyanobacteria was more pronounced compared to algae (Tukey *post hoc*, *p* < 0.05; Figure 2 and Supplementary Table 3) suggesting a higher flexibility of cyanobacteria PSII. There were no differences in effect sizes of a taxon or the experimental medium on the estimated ETR_max_ (Figure 2, see Supplementary Table 4 for details).

## Discussion

A plethora of adaptive responses to predation has been described in microscopic aquatic producers. Although energetic costs of these responses are expected, with their consequences going far beyond an individual, like a role in shaping phytoplankton communities, their underlying molecular and metabolic mechanisms are not fully known. We approached this gap by investigating the primary energy conversion end of the metabolic pathways. One rationale for this focus is the expected responsiveness of the light-dependent photosynthetic processes, as their flexible strategies are regarded as key to successful dominance within phytoplankton communities (Fisher et al., 2020). Indeed, photosystem II (PSII) responds to abiotic stressors: it is vulnerable to excessive radiation (Beecraft et al., 2019), and toxic effects of various anthropogenic substances (Grzesiuk et al., 2016), responds to temperature changes and its efficiency tends to decrease under a variety of stressors: nutrient limitation, reactive oxygen species or high light intensity (Li et al., 2021). Furthermore, PSII electron flow correlates strongly with carbon fixation rate and growth rate in algae, providing a good indicator of stress and costs incurred. Its responses to biotic stresses are less known, though.

We hypothesized that cues of predation elicit changes in the activity of photosynthetic machinery in phytoplankton cells and we applied fluorometry to measure the photosynthetic response of several algal and cyanobacterial species experimentally exposed to chemical cues of a microcrustacean herbivore, *Daphnia*—its presence alone (kairomone) or together with cues of its predatory activity (kairomone + alarm cues). Across several prey taxa and predator-diet contexts we found all: an increase, a decrease, or no change in the operating efficiency of PSII, measured as its quantum yield, and in the maximum electron transport rate (ETR_max_). In most algal and cyanobacterial species studied here, the yield of PSII increased in response to predator-fed *Synechococcus* (kairomone + cyanobacterial alarm cues), whereas in most of these species the yield did not change in response to predator-fed *Acutodesmus* (kairomone + algal alarm cues). Also, cyanobacteria tended not to respond to a non-feeding predator (kairomone alone). The modal qualitative responses of approximated ETR_max_ were similar to those of yield, with fewer reactions to cyanobacterial and more to algal alarm cues.

Previous reports are scarce and, to our best knowledge, the results presented here are the broadest scan of PSII responses in the predation context so far. Lürling and Van Donk (2000) found no differences in PSII efficiency between undefended unicellular and defended colonial populations of green alga *Acutodesmus acutus* upon long-term exposure to an algal-fed herbivore, *Daphnia magna*, the same species as used here. This stays in line with the modal phytoplankton response to algal-fed *Daphnia* in our study: no change in PSII efficiency. More recently, Albini et al. (2019) found decreased PSII efficiency in the green alga *Acutodesmus obliquus* upon long-term exposure to either algal-fed or non-feeding *D. magna*. This report is also consistent with our findings for these particular species under short-term exposure, though the slight mean decrease of PSII efficiency upon exposure to non-feeding *Daphnia* was not significant in our study (Figure 1, top). We tested immediate response to a predator, several algal species, and several species of another taxon, cyanobacteria, in contrast to the previously mentioned studies.

The immediate, i.e., observed here within an hour of exposure, increase or decrease in photosystem efficiency may indicate mobilization or distress, respectively. On one hand, the expression of an adaptive plastic anti-predator response incurs energetic costs, and intensifying photosynthetic processes may be a way to meet the demand. On the other hand, the stress associated with perceived predation presence may be a part of the physiological response and impaired photosynthetic processes may be the resulting cost itself. Somewhat similar, on the katabolic end, the stress induced by predator presence is often associated with the “flight or fight” response demanding mobilization of energy and resulting in increased metabolic rate (Robison et al., 2018). However, a decrease in metabolic rate is also frequently observed. Opposite responses observed in Daphnia when exposed to various predation cues as prey (Pestana et al., 2013; Robison et al., 2018).

In our study, phylogeny appeared to be more important than morphology for photosystem response. We did not observe consistent differences in reaction between defended filamentous and undefended unicellular morphs (as exposures were short, no time was given for other defenses to be functionally expressed). Rather, cyanobacteria tended to increase PSII activity in more trials than algae did, possibly suggesting their overall greater adaptive flexibility of photosynthetic processes in the face of predation. Also, in terms of the absolute strength of the response, the yield of PSII changed more in cyanobacteria in response to kairomone with algal alarm cues (i) than to kairomone alone, and (ii) than in the algae themselves. In general, photosystem activity appeared to be more flexible in response to predation in cyanobacteria than in algae. We are here aware that PSII quantum yield values themselves are argued not to be a proper measure for comparison of the overall photosynthetic efficiency between cyanobacteria and algae (Schuurmans et al., 2015), and we only consider the magnitude and direction of its change.

There are a few possible shortcomings in the methods used in this study, limiting the scope of interpretation of the results. To unify procedures and maximize overall performance, the phytoplankton culturing conditions were standardized according to the procedures commonly used in the laboratory of the Institute for Biochemistry and Biology (University of Potsdam, Germany). Also, we controlled the populations for nutrient limitation before experiments. Yet, we did not fully exclude conditions of sub-optimality in other factors. Also, our estimation of ETR_*max*_ from extrapolated curves might be sub-optimal and should be treated as an approximation. The use of effect sizes overcomes the shortcomings of both the direct effects of environmental conditions on absolute values of measured photosynthetic parameters and the approximation character of the values. However, it does not overcome the potential effect of environmental conditions on the response to predation itself, which to some extent might have affected the results.

The broad scan of responses studied here provides insight into the general trends and the variety of possible reactions, as well as a starting point for future in-depth analyses. One interesting point for future research on the underlying mechanisms would be to follow the dynamics of the response through time, both on a finer time scale and with a broader scope than our pilot tests (Supplementary Table 1E). The rapidity of the response observed here — the time frame being of minutes or dozens of minutes—is meaningful and calls for further elucidation. Another important issue would be to test the generality of the response to algal versus cyanobacterial alarm cues on one hand, as we tested here only one species of each used to feed the predator, or to different predators. This was beyond the scope of our study.

Phytoplankton adjusts their cell phenotype in a changing environment, and flexible energy management may involve physiological photosynthetic and respiratory responses, and adjustments of the molecular machinery responsible for nutrient uptake (Bonachela et al., 2011), or even subcellular architecture (Uwizeye et al., 2021). Still, research on photosynthesis alterations under a biotic stress such as predation is limited (but see e.g., Deore et al., 2020 for reduced non-photochemical quenching proposed as a useful early indicator of predation). We focus here on a single process only, and two related parameters, yet they are recognized as important indicators of phytoplankton cell state. Photosystem II efficiency (either maximum or operating) is regarded as a sensitive physiological parameter that directly reflects phytoplankton growth potential—was even proposed as a measure for forecasting cyanobacterial blooms (Wang et al., 2018). Here we show its responses to a biotic stress—predation.

## Data availability statement

The raw data supporting the conclusions of this article will be made available by the authors, without undue reservation.

## Author contributions

JP proposed the conception of the study. MG, JP, and AW contributed to its design. MG carried out the experiments. AW and JP supervised the experimental work. BP performed the statistical analyses, interpreted the results, and prepared the figures. BP and MG wrote the first draft of the manuscript. AW wrote sections of the manuscript. All authors contributed to the manuscript edition and revision and approved the submitted version.

## References

[B1] Albini D. Fowler M. S. Llewellyn C. Tang K. W. (2019). Reversible colony formation and the associated costs in *Scenedesmus* obliquus. *J. Plankton Res.* 41 419–429. 10.1093/plankt/fbz032

[B2] Baker N. R. (2008). Chlorophyll fluorescence: a probe of photosynthesis In vivo. *Annu. Rev. Plant Biol.* 59 89–113. 10.1146/annurev.arplant.59.032607.092759 18444897

[B3] Beecraft L. Watson S. B. Smith R. E. H. (2019). Innate resistance of PSII efficiency to sunlight stress is not an advantage for cyanobacteria compared to eukaryotic phytoplankton. *Aquat. Ecol.* 53 347–364.

[B4] Bonachela J. A. Raghib M. Levin S. A. (2011). Dynamic model of flexible phytoplankton nutrient uptake. *PNAS* 108 20633–20638. 10.1073/pnas.1118012108 22143781 PMC3251133

[B5] Cerbin S. Wejnerowski Ł Dziuba M. (2013). Aphanizomenon gracile increases in width in the presence of Daphnia. A defence mechanism against grazing? *J. Limnol.* 72 505–511. 10.4081/jlimnol.2013.e41

[B6] Chivers D. P. Mathiron A. Sloychuk J. R. Ferrari M. C. O. (2015). Responses of tadpoles to hybrid predator odours: strong maternal signatures and the potential risk/response mismatch. *Proc. R. Soc. B.* 282:20150365. 10.1098/rspb.2015.0365 26041358 PMC4590445

[B7] Colbourne J. K. Pfrender M. E. Gilbert D. Thomas W. K. Tucker A. Oakley T. H. (2011). The ecoresponsive genome of Daphnia pulex. *Science* 331 555–561. 10.1126/science.1197761 21292972 PMC3529199

[B8] Deore P. Karthikaichamy A. Beardall J. Noronha S. (2020). Non-photochemical quenching, a non-invasive probe for monitoring microalgal grazing: an early indicator of predation by Oxyrrhis marina and Euplotes sp. *Appl. Phycol.* 1 20–31. 10.1080/26388081.2019.1651218

[B9] Eilers P. H. C. Peeters J. C. H. (1988). A model for the relationship between light intensity and the rate of photosynthesis in phytoplankton. *Ecol. Model* 42 199–215. 10.1016/0304-3800(88)90057-9

[B10] Fisher N. L. Campbell D. A. Hughes D. J. Kuzhiumparambil U. Halsey K. H. Ralph P. J. (2020). Divergence of photosynthetic strategies amongst marine diatoms. *PLoS One* 15:e0244252. 10.1371/journal.pone.0244252 33370327 PMC7769462

[B11] Fisher R. M. Bell T. West S. A. (2016). Multicellular group formation in response to predators in the alga Chlorella vulgaris. *J. Evol. Biol.* 29 551–559. 10.1111/jeb.12804 26663204

[B12] Fox J. Weisberg S. (2019). *An R Companion to Applied Regression*, 3rd Edn. Thousand Oaks, CA: Sage.

[B13] Gliwicz Z. M. (2003). Between hazards of starvation and risk of predation: the ecology of offshore animals. *Excell. Ecol.* 12 XXIII–379.

[B14] Grzesiuk M. Wacker A. Spijkerman E. (2016). Photosynthetic sensitivity of phytoplankton to commonly used pharmaceuticals and its dependence on cellular phosphorus status. *Ecotoxicology* 25 697–707. 10.1007/s10646-016-1628-8 26894612

[B15] Harvey E. L. Menden-Deuer S. (2012). Predator-induced fleeing behaviors in phytoplankton: a new mechanism for harmful algal bloom formation? *PLoS One* 7:e46438. 10.1371/journal.pone.0046438 23029518 PMC3460921

[B16] Hessen D. O. Van Donk E. (1993). Morpholigical changes in Scenedesmus induced by substances released from Daphnia. *Arch. Hydrobiol.* 127 129–140. 10.1127/archiv-hydrobiol/127/1993/129

[B17] Hothorn T. Bretz F. Westfall P. (2008). Simultaneous inference in general parametric models. *Biom. J.* 50 346–363. 10.1002/bimj.200810425 18481363

[B18] Jang M.-H. Ha K. Joo G.-J. Takamura N. (2003). Toxin production of cyanobacteria is increased by exposure to zooplankton. *Freshw. Biol.* 48 1540–1550. 10.1046/j.1365-2427.2003.01107.x

[B19] Lampert W (2011). Daphnia: development of a model organism in ecology and evolution. *Excell. Ecol.* 21 XIX+250.

[B20] Li Z. Li W. Zhang Y. Hu Y. Sheward R. Irwin A. J. (2021). Dynamic photophysiological stress response of a model diatom to ten environmental stresses. *J. Phycol.* 57 484–495. 10.1111/jpy.13072 32945529

[B21] Long J. D. Smalley G. W. Barsby T. Anderson J. T. Hay M. E. (2007). Chemical cues induce consumer-specific defenses in a bloom-forming marine phytoplankton. *PNAS* 104 10512–10517. 10.1073/pnas.0611600104 17563379 PMC1965544

[B22] Lürling M. (2003). Phenotypic plasticity in the green algae *Desmodesmus* and *Scenedesmus* with special reference to the induction of defensive morphology. *Ann. Limnol. Int. J. Lim.* 39 85–101. 10.1051/limn/2003014

[B23] Lürling M. (2021). Grazing resistance in phytoplankton. *Hydrobiologia* 848 237–249. 10.1007/s10750-020-04370-3

[B24] Lürling M. Van Donk E. (2000). Grazer-induced colony formation in Scenedesmus: are there costs to being colonial? *Oikos* 88 111–118. 10.1034/j.1600-0706.2000.880113.x 11841302

[B25] Mangiafico S. (2020). *rcompanion: Functions to Support Extension Education Program Evaluation. R Package Version 2.3.25.* Vienna: R Core Team.

[B26] Marzetz V. Spijkerman E. Striebel M. Wacker A. (2020). Phytoplankton community responses to interactions between light intensity, light variations, and phosphorus supply. *Front. Environ. Sci.* 8:539733. 10.3389/fenvs.2020.539733

[B27] Pančić M. Kiørboe T. (2018). Phytoplankton defence mechanisms: traits and trade-offs. *Biol. Rev.* 93 1269–1303. 10.1111/brv.12395 29356270

[B28] Parysek M. Pietrzak B. (2021). Weak swimming response of a bdelloid rotifer to chemical cues of a native copepod predator. *J. Ethol.* 39 135–139. 10.1007/s10164-020-00676-w

[B29] Pestana J. L. T. Baird D. J. Soares A. M. V. A. (2013). Predator threat assessment in Daphnia magna: the role of kairomones versus conspecific alarm cues. *Mar. Freshw. Res.* 64 679–686. 10.1071/MF13043

[B30] Pinheiro J. Bates D. DebRoy S. Sarkar D. R Core Team. (2019). *nlme: Linear and Nonlinear Mixed Effects Models*. Available at: https://CRAN.R-project.org/package=nlme

[B31] Porter K. G. (1976). Enhancement of algal growth and productivity by grazing zooplankton. *Science* 192 1332–1334. 10.1126/science.192.4246.1332 17739838

[B32] R Core Team (2021). *R: A Language and Environment for Statistical Computing.* Vienna: R Foundation for Statistical Computing.

[B33] Robison A. L. Chapman T. Bidwell J. R. (2018). Predation cues influence metabolic rate and sensitivity to other chemical stressors in fathead minnows (*Pimephales Promelas*) and Daphnia pulex. *Ecotoxicology* 27 55–68. 10.1007/s10646-017-1870-8 29101637

[B34] Rojo C. Segura M. Rodrigo M. A. Salazar G. (2009). Factors controlling the colonial structure of *Pediastrum* tetras (*Chlorophyceae*). *Hydrobiol* 617 143–155. 10.1007/s10750-008-9542-6

[B35] Schuurmans R. M. van Alphen P. Schuurmans J. M. Matthijs H. C. P. Hellingwerf K. J. (2015). Comparison of the photosynthetic yield of cyanobacteria and green algae: different methods give different answers. *PLoS One* 10:e0139061. 10.1371/journal.pone.0139061 26394153 PMC4578884

[B36] Selander E. Jakobsen H. H. Lombard F. Kiørboe T. (2011). Grazer cues induce stealth behavior in marine dinoflagellates. *PNAS* 108 4030–4034. 10.1073/pnas.1011870108 21368128 PMC3054020

[B37] Senft-Batoh C. D. Dam H. G. Shumway S. E. Wikfors G. H. (2015). A multi-phylum study of grazer-induced paralytic shellfish toxin production in the dinoflagellate Alexandrium fundyense: a new perspective on control of algal toxicity. *Harmful Algae* 44 20–31. 10.1016/j.hal.2015.02.008

[B38] Shelly K. Holland D. Beardall J. (2010). “Assessing nutrient status 955 of microalgae using chlorophyll a fluorescence,” in *Chlorophyll a Fluorescence in Aquatic Sciences: Methods and Applications*, eds Suggett D. J. Borowitzka M. A. Pra ìsil O. (Heidelberg: Springer), 223–235. 10.1007/978-90-481-9268-7_11

[B39] Spijkerman E. Stojkovic S. Holland D. Lachmann S. Beardall J. (2016). Nutrient induced fluorescence transients (NIFTs) provide a rapid measure of P and C (co-)limitation in a green alga. *Eur J Phycol* 51 47–58. 10.1080/09670262.2015.1095355

[B40] Torchiano M. (2020). *effsize: Efficient Effect Size Computation.* Vienna: R Core Team, Available online at: https://CRAN.R-project.org/package=effsize.

[B41] Uwizeye C. Decelle J. Jouneau P. H. (2021). Morphological bases of phytoplankton energy management and physiological responses unveiled by 3D subcellular imaging. *Nat. Commun.* 12:1049. 10.1038/s41467-021-21314-0 33594064 PMC7886885

[B42] Van Donk E. Ianora A. Vos M. (2011). Induced defences in marine and freshwater phytoplankton: a review. *Hydrobiologia* 668 3–19. 10.1007/s10750-010-0395-4

[B43] Wang H. Zhu R. Zhang J. Ni L. Y. Shen H. Xie P. (2018). A novel and convenient method for early warning of algal cell density by chlorophyll fluorescence parameters and its application in a highland lake. *Front. Plant Sci.* 9:13. 10.3389/fpls.2018.00869 30002664 PMC6031977

[B44] Wejnerowski Ł Cerbin S. Wojciechowicz M. Jurczak T. Glama M. Meriluoto J. (2018). Effects of Daphnia exudates and sodium octyl sulphates on filament morphology and cell wall thickness of *Aphanizomenon* gracile (Nostocales), Cylindrospermopsis raciborskii (Nostocales) and Planktothrix agardhii (Oscillatoriales). *Eur. J. Phycol.* 53 280–289. 10.1080/09670262.2018.1442585

[B45] Wiedner C. Rücker J. Brüggemann R. Nixdorf B. (2007). Climate change affects timing and size of populations of an invasive cyanobacterium in temperate regions. *Oecologia* 152 473–484. 10.1007/s00442-007-0683-5 17375336

[B46] Yang Z. Kong F. X. Yang Z. Zhang M. Yu Y. Qian S. Q. (2009). Benefits and costs of the grazer-induced colony formation in Microcystis aeruginosa. *Ann. Limnol. Int. J. Limnol.* 45 203–208. 10.1051/limn/2009020 26224387

[B47] Yokota K. Sterner R. W. (2011). Trade-offs limiting the evolution of coloniality: ecological displacement rates used to measure small costs. *Proc. R. Soc. B Biol. Sci.* 278 458–463. 10.1098/rspb.2010.1459 20739317 PMC3013416

