# Supplementary material for: Photosynthetic activity in both algae and cyanobacteria changes in response to cues of predation
Source: Front Plant Sci. 2022 Jul 25;13:907174. doi: 10.3389/fpls.2022.907174 (PMC9358279; doi:10.3389/fpls.2022.907174)
Supplement: Supplementary file 1 [file Data_Sheet_1.docx]

**Supplementary Material & Figures**

**NIFT measurements**

Nutrient Induced Fluorescence Transient (NIFT) is the change in the chlorophyll a (chl a) fluorescence observed when nutrient-limited producer is exposed to a pulse of the limiting nutrient and provides a way of testing for the limiting nutrient in phytoplankton populations (Spijkerman et al. 2016). We measured NIFTs in all studied algae and cyanobacteria cultures to check if they were limited by inorganic phosphorus (P_i_) or/and nitrogen (N). We used a Phyto-PAM fluorometer (Heinz Walz GmbH, Effeltrich, Germany) and recorded the chl a fluorescence (Ft) every 3 s without the application of a saturating pulse (Phytowin_v1.47). The actinic light source in the measuring cuvette was set to 120 μmol photons m^–2^ s^–1^. Chl a fluorescence was first recorded for at least 1 min to obtain a stable value, after which the response to a spike of both P_i_ and N was subsequently monitored for several minutes. For P_i_-addition we used a final standard concentration of 10 μM KH_2_PO_4_. For N-addition we used a final standard concentration of 100 μM (NH)_4_SO_4_. The decrease in Ft following the spike of a limiting nutrient (NIFT) would confirm the P_i_ or N limitation of cultures. The absence of such response after the nutrients spike shows that the algae and cyanobacteria are not limited by nitrogen or phosphorus (Supplementary Figure 1A). The results of NIFT measurements are shown in Supplementary Figure 1B and C.


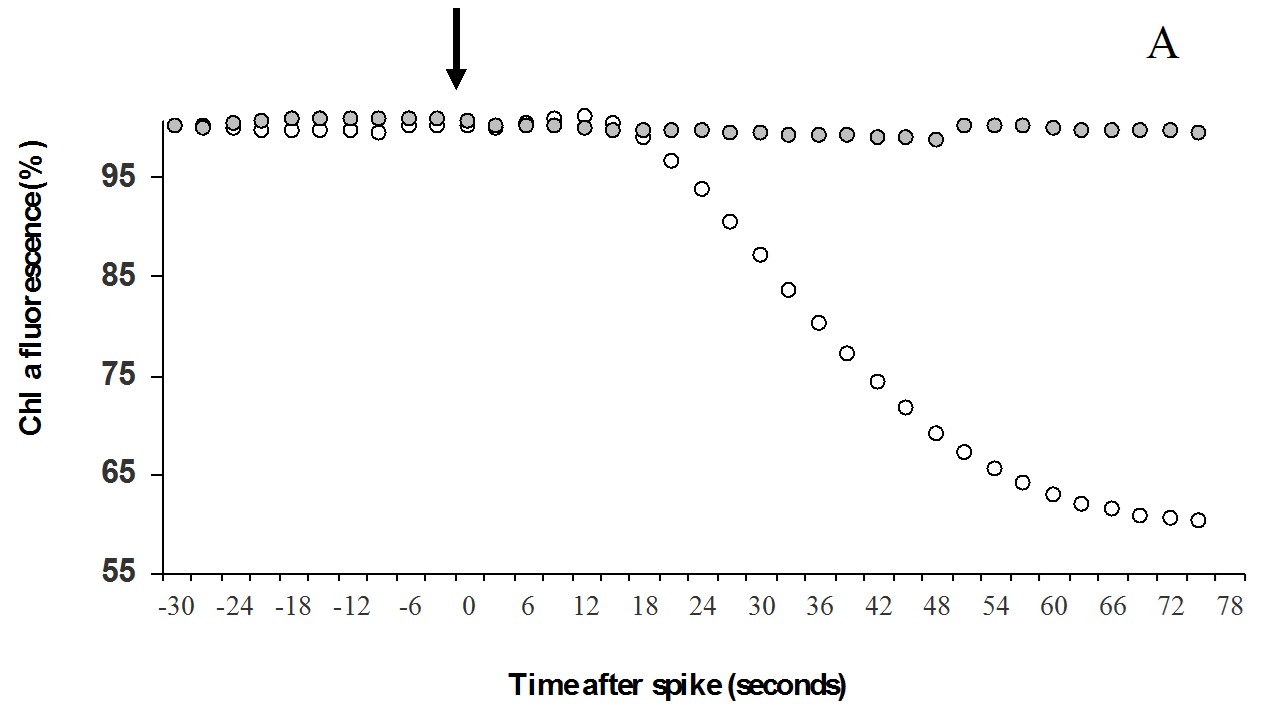


Supplementary Figure 1A. An example of nutrient replete (full circles) and nutrient limited (empty circles) *Acutodesmus obliquus* response after a nutrient spike (see Suppl. Material in Grzesiuk et al. 2016)

Supplementary Figure 1B. NIFT measurements in experimental algae cultures: chl a response after a P_i_ and N spike (↓).

Supplementary Figure 1C. NIFT measurements in experimental cyanobacteria cultures: chl a response after a P_i_ and N spike (↓).

References:

Grzesiuk M., Wacker A., Spijkerman E., Photosynthetic sensitivity of phytoplankton to commonly used pharmaceuticals and its dependence on cellular phosphorus status. Ecotoxicology 25:697-707

Spijkerman E, Stojkovic S, Holland D, Lachmann SC & Beardall J. 2016. Nutrient induced fluorescence transients (NIFTs) provide a rapid measure of P and C (co-)limitation in a green alga, European Journal of Phycology, 51:1, 47-58, DOI: 10.1080/09670262.2015.1095355
