# Supplementary material for: Photosynthetic activity in both algae and cyanobacteria changes in response to cues of predation
Source: Front Plant Sci. 2022 Jul 25;13:907174. doi: 10.3389/fpls.2022.907174 (PMC9358279; doi:10.3389/fpls.2022.907174)
Supplement: Supplementary file 2 [file Data_Sheet_2.docx]

Supplementary Table 1A. The F’ (first flash, relative units), Fm’ (first flash, relative units), Yield, alpha (relative units), ETR_max_ (µmol electrons m^-2^ s^-1^), I_K_ (µmol photons m^-2^ s^-1^), of different algae species*,* after a 10 minute exposure to *Daphnia* water or in control, Phyto-PAM readings at four channels (1-4).

Supplementary Table 1B. The F’ (first flash, relative units), Fm’ (first flash, relative units), Yield, alpha (relative units), ETR_max_ (µmol electrons m^-2^ s^-1^), I_K_ (µmol photons m^-2^ s^-1^), of different cyanobacteria species*,* after a 10 minute exposure to *Daphnia* water or in control, Phyto-PAM readings at four channels (1-4).

Supplementary Table 1C. The F’ (first flash, relative units), Fm’ (first flash, relative units), Yield, alpha (relative units), ETR_max_ (µmol electrons m^-2^ s^-1^), I_K_ (µmol photons m^-2^ s^-1^), of different algae species*,* after a 60 minute exposure to *Daphnia* water or in control, Phyto-PAM readings at four channels (1-4).

Supplementary Table 1D. The F’ (first flash, relative units), Fm’ (first flash, relative units), Yield, alpha (relative units), ETR_max_ (µmol electrons m^-2^ s^-1^), I_K_ (µmol photons m^-2^ s^-1^), of different cyanobacteria species*,* after a 60 minute exposure to *Daphnia* water or in control, Phyto-PAM readings at four channels (1-4).

Supplementary Table 1E. The F’ (first flash, relative units), Fm’ (first flash, relative units), Yield, alpha (relative units), ETR_max_ (µmol electrons m^-2^ s^-1^), I_K_ (µmol photons m^-2^ s^-1^), of *Acutodesmus obliquus,* after a 10, 60, 120,240 minute exposure to *Daphnia* water or in control, Phyto-PAM readings at four channels (1-4).
