# Supplementary material for: Photosynthetic activity in both algae and cyanobacteria changes in response to cues of predation
Source: Front Plant Sci. 2022 Jul 25;13:907174. doi: 10.3389/fpls.2022.907174 (PMC9358279; doi:10.3389/fpls.2022.907174)
Supplement: Supplementary file 3 [file Data_Sheet_3.docx]

Supplementary Table 2 Analysis of deviance table (type II tests) for GLS model on PS II operating efficiency absolute effect size in algae and cyanobacteria exposed in grazer media treatments.

|  | df | *Chi^2^* | P |
| --- | --- | --- | --- |
| taxon (algae vs. cyanobacteria) | 1 | 0.7485 | 0.3870 |
| medium treatment | 2 | 6.9373 | * 0.0312 |
| taxon x medium treatment | 2 | 11.0777 | ** 0.0039 |

Supplementary Table 3 Multiple comparisons of means: Tukey contrasts for GLS model on PS II operating efficiency absolute effect size in algae (A) and cyanobacteria (C) exposed in grazer media treatments: *Daphnia* fed algae (alg), cyanobacteria (cya) or unfed (hun).

|  | estimate | SE | z | P |
| --- | --- | --- | --- | --- |
| A.cya - A.alg | 0.0256 | 0.3597 | 0.0711 | 1.0000 |
| A.hun - A.alg | -0.2494 | 0.3205 | -0.7782 | 0.9640 |
| C.alg - A.alg | 2.0844 | 0.7485 | 2.7846 | * 0.0473 |
| C.cya - A.alg | 2.0867 | 1.0418 | 2.0030 | 0.2943 |
| C.hun - A.alg | -0.4133 | 0.2218 | -1.8633 | 0.3748 |
| A.hun - A.cya | -0.2750 | 0.3971 | -0.6925 | 0.9783 |
| C.alg - A.cya | 2.0588 | 0.7844 | 2.6247 | 0.0726 |
| C.cya - A.cya | 2.0611 | 1.0678 | 1.9302 | 0.3353 |
| C.hun - A.cya | -0.4389 | 0.3228 | -1.3599 | 0.7107 |
| C.alg - A.hun | 2.3338 | 0.7672 | 3.0418 | * 0.0217 |
| C.cya - A.hun | 2.3361 | 1.0553 | 2.2138 | 0.1939 |
| C.hun - A.hun | -0.1639 | 0.2784 | -0.5888 | 0.9895 |
| C.cya - C.alg | 0.0023 | 1.2535 | 0.0019 | 1.0000 |
| C.hun - C.alg | -2.4977 | 0.7315 | -3.4143 | ** 0.0066 |
| C.hun - C.cya | -2.5000 | 1.0296 | -2.4281 | 0.1197 |

Supplementary Table 4 Analysis of deviance table (type II tests) for GLS model on ETRmax absolute effect size in algae and cyanobacteria exposed in grazer media treatments.

|  | df | *Chi^2^* | P |
| --- | --- | --- | --- |
| taxon (algae vs. cyanobacteria) | 1 | 1.3059 | 0.2531 |
| medium treatment | 2 | 2.8722 | 0.2379 |
| taxon x medium treatment | 2 | 2.9408 | 0.2298 |
